# Understanding Risk Perception toward Food Safety in Street Food: The Relationships among Service Quality, Values, and Repurchase Intention

**DOI:** 10.3390/ijerph18136826

**Published:** 2021-06-25

**Authors:** Kyung Hwa Seo, Jee Hye Lee

**Affiliations:** 1 Department of Hotel Culinary Arts and Bakery, Ulsan College, 101 Bongsu-ro, Dong-gu, Ulsan 44022, Korea; khseo@uc.ac.kr; 2 Department of Food & Nutrition, College of Human Ecology, University of Ulsan, 93 Daehak-ro, Ulsan 44610, Korea

**Keywords:** street food, service quality, utilitarian value, hedonic value, risk perception

## Abstract

This paper aims to identify service quality dimensions of street food that have an impact on utilitarian and hedonic values and to determine the effect of utilitarian and hedonic values on repurchase intention. It also examines the moderating effect of risk perception toward street food safety on the relationship between service quality and perceived value. An Internet survey was performed in Korea with 285 respondents. The results confirmed that the five dimensions of street food’s service quality—food quality, employee service, physical environment, price, and rapidity of service—had positive impacts on utilitarian and hedonic values. All perceived value (utilitarian, hedonic) has an impact on repurchase intention. Finally, the food quality of street food showed a stronger influence on utilitarian value among the low-risk perception group than the high-risk perception group depending on the consumers’ level of awareness of food safety. This provides new insights for marketing strategies to attract domestic/foreign consumers to street food vendors and for creating a new food culture by emphasizing important domains of service quality, the relation of quality to consumer values, and risk perception toward food safety in street food.

## 1. Introduction

Street food is ready-to-eat food and drinks sold by vendors on the street. Consumers frequently eat street food because it provides convenient, delicious, and cheap meals [1]. Additionally, street food provides benefits to society such as building a local culinary tradition, generating a large source of employment, and enhancing tourism [2]. As such, street food is considered not only a meal replacement for its own people, but also an attractive component of night markets in various Asian countries. Some examples of such attractions are the hawker centers in Singapore, night markets in Taiwan, street stalls in Korea, and mobile food stalls (yatai) in Japan [3]. Street foods are a nearly worldwide phenomenon of urban life from New York City’s hot dog cart to the taco stand of Mexico [4]. Recently, street food prepared in food trucks is evolving street food culture in some places by providing fresh, clean, and delicious food with entertainment [5]. Well-established street food represents unique cultural food traditions and is one way to experience a unique culture and to satisfy the desire to eat a delicious, authentic cuisine [6].

In the world in the 21st century, the revitalization of the street food industry by attracting consumers including local and foreign tourists is important for generating profits. Despite the potential growth of street food, so far, it has not been actively reviewed in street food research. As competition in the restaurant industry becomes more intense, consumers’ expectations for service quality increases, so providing good service quality is a critical strategy for survival in the food market [7,8,9]. Service quality refers to the extent of the discrepancy between consumers’ expectations for service and their evaluation of the performance of that service [10]. Perceived service quality is determined by how consumers evaluate the actual performance compared to what they expected. The higher the evaluation of service quality, the more likely consumers will return, spread favorable word of mouth, and increase brand loyalty [11,12].

Although service quality is one of the critical factors affecting consumer behavior, there has been less attention given to identifying the service quality dimensions of street food [13]. It is difficult to come up with specific strategic methods for sustained performance without understanding the quality of street food. Additionally, understanding which component of service quality is more important is necessary to aid marketers and owners, both of whom need to create the best efficiency and profits based on limited resources. As a result, there is a need to identify determinant dimensions and attributes of service quality that consumers consider important when evaluating their street food experience. There is a consensus that food quality is an important dimension of restaurant experiences regardless of the type of food service [14,15,16]. Additionally, food freshness, appropriate food temperature, presentation, and healthy options are critical attributes in food quality [17,18]. Employee service plays a pivotal role in service encounter. Because employees interact directly with consumers, the quality of their services leads to favorable evaluation, higher satisfaction, and an increase in purchases [19]. Heung and Gu [20] found five environmental qualities to service: facility aesthetic, ambience, spatial layout, view, and employee appearance. Furthermore, price plays an important role in the customer’s purchase decision. Saad Andaleeb and Conway [15] determined that price has a critical effect on satisfaction. The characteristics of street food operation such as easier preparation of foods, low initial investment, lower labor requirements, and lower overhead enable setting a low price for the products. Finally, rapidity is critical in consumer satisfaction [21]. Wu [22] asserted that in quick service restaurants, the speed of service is considered an important attribute in service quality. Thus, in this current study, 5 dimensions and 14 attributes are identified for the service quality of street food based on a review of previous literature: (1) food quality (taste, freshness, and temperature), (2) employee service (staff’s kindness, knowledgeability and confidence, and attentiveness to complaints), (3) physical environment (interior, music, and light), (4) price (reasonable price compared to taste, reasonable price compared to quantity, and economic price), and (5) rapidity (rapid service and time saving).

Furthermore, marketing practitioners and researchers emphasize that creation of value is the key for market success [23], and value plays a critical role in behavioral intention [24]. Perceived value is the consumer’s overall assessment of the utility of service based on perceptions of what is received and what is paid [25], i.e., a trade-off between benefit provided by the service and the cost or sacrifice for acquiring the service [26]. Frondizi [27] argued that when a quality is valued, the association between a particular product/service and a specific consumer is reinforced. Thus, once dimensions and attributes of service quality in street foods have been determined, how the perceived value can be enhanced through these attributes of the experience provides insights for street food vendors and marketers. Although there has been increased attention given to the association between service quality and perceived value [28,29], empirical work conducted to address the effect of determinant dimensions of service quality on perceived value in street food has not yet been adequately addressed. Furthermore, the current study focused on two structural dimensions of perceived value—utilitarian and hedonic value—because more research agrees that perceived value in a service setting is better explained when utilitarian and hedonic values are conceptualized together [30]. Utilitarian value refers to benefits provided by street food consumption compared to what is paid, and hedonic value refers to emotional pleasure derived from street food consumption [31,32,33]. The work of Zeithaml [25] verified the impact of service quality on perceived value. Empirical research found evidence of a positive impact of service quality on perceived value [34,35].

Consumer behavior involves purchasing a product again or recommending it to others according to the customer’s experience. In particular, repurchase intention refers to an individual’s decision to buy again from the same company, taking into account his or her situation [36]. Numerous empirical studies determined perceived value as an important factor for understanding a consumer’s selection process [33] and showed it to be a good predictor for explaining repurchase intention [34,37]. As the consumer perceives higher service quality, the perceived value becomes higher, and this influence is related to repurchase intention. As a result, if consumers assess the service quality of street food positively, the perceived value of street food will also improve, which will lead to a repurchase intention.

Lastly, street foods are associated with food safety issues [38]. The vending of street food is usually performed in small mobile vending units in which there may be a lack of hygiene, such as inappropriate food management (e.g., preparation, storage, handling practices, etc.) and poor equipment and environments [39]. Research has focused on identifying sicknesses resulting from street food consumption, or certain causative agents of foodborne illness associated with street food [40]. However, to understand consumer behavior, an explanation of the negative impact relationship of risk perception toward food safety in street food needs to be further reviewed, because consumers perceive food safety as a critical element when selecting a restaurant [41]. Perceived risk has been described as consumers’ perceptions of the uncertainty and adverse outcome of purchasing a service [42]. The concept of perceived risk most often used by consumer researchers defines risk in terms of the consumer’s perceptions of the uncertainty and adverse consequences of buying a product (or service). Perceived risk is often regarded as an antecedent that negatively affects consumers’ perceived value [43,44]. Chang and Tseng [45] asserted that improving quality and reducing customer shopping risks can improve perceived value. Moreover, consumers’ risk perception also has a negative impact on value formation because risk involves physical loss such as health problems caused by unhygienic foods. This current study assumes that consumer risk perception toward street food safety has a negative impact on perceived value (utilitarian and hedonic values), of which researchers have not yet gained a clear understanding. Street food is involved with food safety issues, from preparation of ingredients to the cooking process to sales. Vendors often lack sanitary conditions due to poor hand washing and inadequately cleaned materials and equipment due to lack of water supply facilities, and it is difficult to maintain the optimum temperature for supplies because of poor infrastructure such as a lack of refrigeration. In other words, this environment can cause foodborne illness due to recontamination, cross-contamination, and spread of pathogens [38], and some of these illnesses can even be fatal. Furthermore, at the point of sale, food items are exposed to pollutants such as dirt, dust, or sand. Thus, this current study proposes that perceived food safety risk toward street food plays a role in moderating the relationship between food quality and perceived value.

Thus, the following hypotheses were proposed.

**Hypothesis** **1.** *Food quality positively affects utilitarian value.*

**Hypothesis** **2.** *Employee service positively affects utilitarian value.*

**Hypothesis** **3.** *Physical environment positively affects utilitarian value.*

**Hypothesis** **4.** *Price positively affects utilitarian value.*

**Hypothesis** **5.** *Rapidity positively affects utilitarian value.*

**Hypothesis** **6.** *Food quality positively affects hedonic value.*

**Hypothesis** **7.** *Employee service positively affects hedonic value.*

**Hypothesis** **8.** *Physical environment positively affects hedonic value.*

**Hypothesis** **9.** *Price positively affects hedonic value.*

**Hypothesis** **10.** *Rapidity positively affects hedonic value.*

**Hypothesis** **11.** *Utilitarian value positively affects repurchase intention.*

**Hypothesis** **12.** *Hedonic value positively affects repurchase intention.*

**Hypothesis** **13.** *Perceived risk moderates the effect of food quality on utilitarian value.*

**Hypothesis** **14.** *Perceived risk moderates the effect of food quality on hedonic value.*

## 2. Materials and Methods

### Sample, Data Collection, and Instrument Development

The data analyzed in this study were collected for consumers in all parts of Korea who had experience purchasing street food within the prior year. We commissioned a professional online survey company to collect data, and 350 questionnaires were distributed via mobile phone and e-mail, and a coupon (USD 5) was given as compensation. Before responding, we explained the confidentiality of their personal information and responses, and they received a response via mobile phone and email. During the period of January 2018, the online surveys offered advantages such as lower costs and faster responses [46]. Out of 350 surveys, 298 questionnaires were returned (response rate: 85.1%), but only 285 questionnaires were used for the analysis due to missing values and biased responses in the survey.

In order to achieve the goals of this study, the concepts of the service qualities of street food, perceived values, and repurchase intention were established on the basis of the existing literature to ensure the validity of the research [47,48,49,50,51,52,53,54]. First, respondents answered all items on the basis of a 7-point scale (1: strongly disagree to 7: strongly agree) regarding the quality of the street food, perceived value, and revisit intention. The service qualities of street food consisted of a total of five elements—food quality, employee service, physical environment, price, and rapidity; the perceived values consisted of two elements—utilitarian and hedonic, and repurchase intention was also included. In addition, the items of risk perception toward street food safety as moderating effect variables were constructed. Finally, there were questions about participant demographics (e.g., gender, age, and purpose of purchase).

Measurement items of service quality of street food were adapted from Back [47], Ryu, and Lee [48], Prayag et al. [49], and Line et al. [50]. The service quality of street food was tested through 14 items regarding service quality: food quality, employee service, physical environment, price, and rapidity. Perceived value was adapted from Hyun et al. [51], Ryu et al. [24], and Kim and Han [52]. The perceived value evaluation was made from six items regarding utilitarian and hedonic value. Repurchase intention was measured with three items adapted from Ryu et al. [53] and Canny [54]. Lastly, three items addressing risk perception toward street food safety were adapted from the previous studies [3,55], including “I suspect that street food uses safe ingredients”, “Street food has poor sanitation management due to lack of water facilities”, and “Street food has unclean environment”.

## 3. Results

### 3.1. Description of Research Sample

The demographic profile of the respondents is shown in Table 1. Respondents consisted of 49.1% male and 50.9% female. In regard to age, 43.2% of the respondents were 20–29 years old, 42.4% were 30–39, and 14.4% were 40 or older. A total of 54.8% of the participants were office workers, 4.9% were self-employed, and 2.8% were government employees. Overall, 82.4% of participants purchased street food 1–5 times per month, 41% got it 6–10 times, and 1.8% got it 11–15 times. For the purpose of their purchase, 73.6% of the respondents (the highest rate) indicated “I eat street foods as a snack”, while 23.2% ate street food as a full meal, and 2.8% ate street food as a tourist activity for experiencing local culture.

### 3.2. Confirmatory Factor Analysis and Reliability Analysis

To assess the reliability of the measurement scales, Cronbach’s alpha was estimated, and in all cases, it was higher than 0.75, which is the threshold (Table 2). Furthermore, the CFA results gave a reference point from which to construct validity tests and provide a better understanding of the measurement results [56]. Based on the CFA results, we analyzed convergent validity, discriminant validity, and reliability of all the multi-items. All indicators loaded on the proposed constructs were significant at *p* < 0.001. Composite construct reliability (CCR) estimates, ranging from 0.850 to 0.933 above the recommended cut-off of 0.70 [57], were acceptable. The average variance extracted (AVE) had to be greater than the 0.50 cut-off for all proposed constructs [58]; results from 0.507 to 0.751 satisfied the requirements. The discriminant validity of the measurement model was evaluated by comparing the squared correlation between each AVE value and two potential factors (Table 3). The measured result of discriminant validity showed from 0.019 to 0.584. It was considered to provide the validity of the concept.

### 3.3. Structural Equation Modeling (SEM)

**Figure 1:**
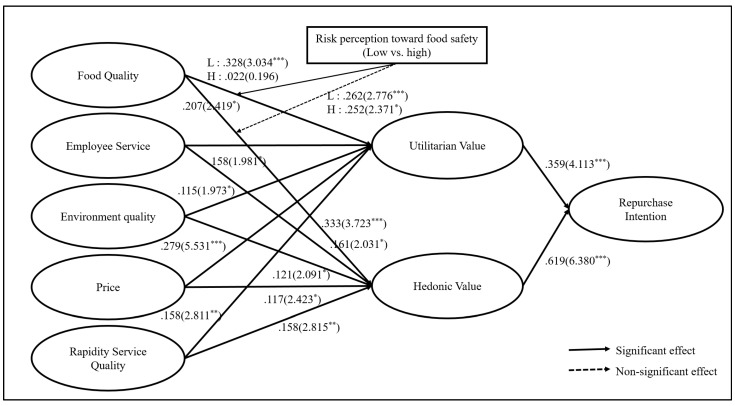
Structural equation model with parameter estimates. Note: standard coefficient (t value) * *p* < 0.05, ** *p* < 0.01, *** *p* < 0.001.

SEM was used to assess the relationship between the potential factors presented as hypotheses. Table 4 explains the result of the estimated model, illustrating the direction and magnitude of the impact of the standardized path coefficients. The standardized chi-squared (χ^2^/degree of freedom) value was 1.850 lower than the cut-off standard of 3.0 [55] and the fit was confirmed as acceptable. Additionally, the chi-square (χ^2^ = 382.969) for this model was statistically significant (*p* < 0.001) with 207 degrees of freedom, and the data of the model showed a good fit. The data for the structural model demonstrated that the other fit indexes also fit reasonably (GFI = 0. 895; NFI = 0.899; CFI = 0.950; RMSEA = 0.055), and it was deemed satisfactory. All the standardized path coefficients are shown together with t-values and results about each hypothesis (see Table 4, Figure 1). They show that all hypotheses are accepted.

H1~H5 were supported. Food quality (β = 0.207; t = 2.419; *p* < 0.05), employee service quality (β = 0.158; t = 1.981; *p* < 0.05), physical environment (β = 0.115; t = 1.973; *p* < 0.05), price (β = 0.279; t = 5.531; *p* < 0.001), and rapidity (β = 0.158; t = 2.811; *p* < 0.01) had a significant effect on utilitarian value. H6~H10 were supported. Food quality (β = 0.333; t = 3.723; *p* < 0.001), employee service quality (β = 0.161; t = 2.031; *p* < 0.05), physical environment (β = 0.121; t = 2.091; *p* < 0.05), price (β = 0.117; t = 2.423; *p* < 0.05), and rapidity (β = 0.158; t = 2.815; *p* < 0.01) had significant effects on hedonic value. The results verified that the service quality of street food in its various dimensions is an important variable affecting utility and hedonic value. H11 and H12 were supported. Utilitarian value (β = 0.359; t = 4.113; *p* < 0.001) and hedonic value (β = 0.619; t = 6.380; *p* < 0.001) had a significant effect on repurchase intention.

### 3.4. Moderating Effects of Risk Perception toward Street Food Safety

To comprehensively measure the moderating effects, the structural invariance assessment aimed to examine whether the proposed structural model is perceived differently between the low-risk (N = 125) and high-risk (N = 165) perception groups (see Table 5). The finding indicated that the high-risk perception group had statistically significant differences, regarding the link between food quality and utilitarian value (△χ^2^ (df = 1) = 5.050 > △χ^2^ (df = 1) = 3.840). The results showed that the effects of food quality on utilitarian value were significantly stronger in the low-risk perception group (β = 0.454) than in the high-risk perception group (β = 0.022). The findings indicated the degree of risk perception significantly moderates the path between food quality of street food and utilitarian value. In other words, as Table 6 shows, the lower the risk degree of food handling is, the greater the influence of the quality of street food on utilitarian value (H13). However, according to the food safety risk group, there was no significant difference in the effect of food quality on hedonic value (△χ^2^ (df = 1) = 0.780 < △χ^2^ (df = 1) = 3.840). Thus, H13 was supported.

## 4. Discussion

Although previous literature has dealt with the food safety issues of street food [38], researchers have made comparatively little effort to examine the issue of service quality, especially the unique attributes of street food and the structural relationship among ‘service quality-perceived value-intention to revisit’ in the context of street foods. The results found that street food’s service quality had positive impacts on perceived value. This finding corroborates earlier research showing the impact of service quality on utilitarian and hedonic value at coffee outlets [30] and at fine dining restaurants [59]. Most of all, the results indicate that a reasonable price is the most important predictor for utilitarian value, and food quality is the most significant predictor for hedonic value. Kwon and Jain [60] stated that low price and convenience are utilitarian shopping benefits.

This study also found positive influences of perceived utilitarian and hedonic value on repurchase intention. This finding corroborates the results of Ryu et al. [24], which showed that perceived value and hedonic value of the fast-casual dining experience have a positive impact on behavioral intentions. Typical street food offers foods at a low price in an efficient manner; therefore, utilitarian value is critical for the repurchase intention of street food. It indicates that the functional utilitarian aspects of consumer value are important predictors of repurchase intention; in addition, hedonic aspects of consumer value play a significant role in positive behavioral intentions in the context of street food. That is, the eating experience of street food might be aptly described as emotional-oriented behavior. For example, watching the process of cooking or eating outdoors—which is a bit of a different atmosphere from eating indoors—offers an enjoyable activity. Sometimes hedonic experience is maximized on special occasions such as traveling or attending festivals; as such, enhancing hedonic value leads to an improvement in behavioral intentions.

Our analysis found that the food quality of street food showed a stronger effect on utilitarian value, varying depending on the level of perceived risk toward food safety (low vs. high). In other words, the food quality of street food showed a stronger influence on utilitarian value among the low-risk perception group of consumers than the high-risk perception group. It indicates that removing the riskier aspects of street food can improve utilitarian value, thereby increasing repurchase intention. This is similar to the findings of Chang and Tseng [45]. However, no moderating effect of risk perception and food quality on hedonic value was found. As stated earlier, this study result indicated that food quality is very important in the perception of hedonic value. It emphasizes the significant role of food quality in hedonic value. If food quality is perceived to be higher than perceived risk of food safety, consumers can fully enjoy the street food.

The theoretical implications of this study are as follows. First, this study identifies the essential five dimensions in service quality of street foods. Although previous researchers suggested various types of service quality according to restaurant classification, there is a lack of service quality research in the context of street food, so there is a limitation in suggesting detailed marketing strategies for street food. This study is meaningful for extending the theoretical scope of existing research by presenting the validity of measurement factors related to the service quality of street food.

Secondly, little attention has been paid to examining the structural relationship of ‘service quality–perceived value–repurchase intention’ in the field of street food. The service quality of street food has been distinguished from other types of industry (i.e., restaurant or hotel) or food service operations (fast or luxury restaurant), so examining the structural relationship in the street food context contributes to the field of tourism and hospitality, since its potential is valued highly as a tourism product. In addition, the finding provides a better explanation about perceived value by examining two structural dimensions (utilitarian and hedonic value) unlike previous research, which has focused on the perceived value with only one dimension.

Thirdly, the food safety issues of street food have been consistently pointed out, but the findings confirmed that there are differences in the influence of food quality on utilitarian value depending on the level of perceived risk toward street food. The influence of food quality on utilitarian value was lower among consumers in the high-risk perception group than those in the low-risk perception group. This finding emphasizes the importance of researching the role of risk perception in the structural relationship.

The managerial implications are as follows. First, the findings showed that all five attributes of street food service quality play an important role in utilitarian and hedonic value. In order to improve food quality and employee service, the following marketing strategies are suggested: constant menu development and evaluation, regular monitoring for food quality management, and education for better service. Because local governments pay attention to street food as a tourism product that can revitalize the local economy, these governments should provide service manuals for operation of street food management and implement education that can foster employees’ knowledge about food and services. Consumers prefer pleasant and clean environments, so creating a place in which street food can be fully enjoyed while demonstrating good kitchen hygiene is a priority to be managed. Many consumers expect a low price when they purchase street food, and the price is even more important when the target segmentation is low-income consumers. Besides that, street food with rapid service can be more competitive for busy, modern people. Street food vendors should serve food more quickly by utilizing already prepared ingredients, minimizing cooks’ movement, monitoring serving time, or continually developing new menus for rapid service.

Second, this study confirmed the association between perceived value and repurchase intention, which has not been explored enough in the context of street food. Marketing activities in street food should focus on producing a more enjoyable and pleasant experience (e.g., entertaining environment, rapid service, kind employees). In particular, the finding that hedonic value affects repurchase intention of street food indicates the potential of street food as a tourism product. If street food in a festival or night market provides fun or enjoyment, it can lead to an increase in repurchasing of street foods by improving the hedonic value to consumers.

Third, the finding showed the important role of risk perception on the relationship between food quality and perceived value. Thus, street food vendors should make an effort to minimize risks in order to satisfy consumers’ various demands for street food safety (e.g., clean environment, refrigeration facilities, water supply facilities, and storing and cooking food under hygienic conditions) so that street food businesses can change the existing expectation that street food is unsafe. Local governments need to strengthen supervision of distributors so that safe food materials can be supplied, and they also should periodically conduct sanitary inspections (including food poisoning tests) of street food businesses. Additionally, street food vendors should constantly check to ensure that food materials are stored well at appropriate temperatures. Lastly, providing information such as the food safety certification of food materials could reduce the consumers’ perceived risk.

Although this study makes several contributions to street food literature, it has some limitations. The distribution of respondents’ demographic and socioeconomic characteristics was not equal. Furthermore, using convenience sampling can have some limitations to the generalizability of the findings, and the low response rate (85.1%) was a limitation of this current study. Future studies should re-examine our research questions with an equal sample size and with more diverse respondents from various countries. Additionally, investigating comparisons of consumers’ risk perception toward food safety between countries where food hygiene supervision is well conducted and countries where it is not well conducted might be interesting.

## 5. Conclusions

Consequently, this study confirms the impact of five dimensions in service quality—food quality, employee service, physical environment, price, and rapidity—on perceived utilitarian and hedonic value, and, in turn, the perceived value directly improves intention to repurchase. Most of all, the results indicate that reasonable price is the most important predictor on utilitarian value, and food quality is the most significant predictor on hedonic value.

This study also found regarding the impact of service quality on utilitarian value, the group that had lower risk perception showed a greater effect on utilitarian value compared to the group that perceived street food as riskier. It indicates that removing risk aspects of street food can improve perceived utilitarian value, thereby increasing repurchase intention. It provides new insights for marketing strategies to attract domestic/foreign consumers to street food vendors and for creating a new food culture by emphasizing important domains of service quality and its relation to consumer values.

## Figures and Tables

**Table 1:** Demographic characteristics of respondents (*n* = 285).

	Characteristics	N	%
Gender	Male	140	49.1
	Female	145	50.9
Age	20–29	123	43.2
	30–39	121	42.4
	40+	41	14.4
Occupation	Office worker	56	54.8
	Self-employed	14	4.9
	Government employees	8	2.8
	Housewife	14	4.9
	Production employee	6	2.1
	Students	50	17.5
	Unemployed	29	10.2
	Other	8	2.8
Frequency of street food purchase	1–5	235	82.4
(per month)	6–10	41	14.4
	11–15	5	1.8
	16–20	2	0.7
	21–30	2	0.7
Purpose of purchase	Snack	210	73.6
	As full meal	66	23.2
	Food tour	8	2.8
	Others	1	0.4

**Table 2:** Reliabilities and confirmatory factor analysis for the model.

Construct	Stand. Loadings (*t*-Value)	CCR	AVE	Cronbach’s Alpha
**Food quality** (3.49 ± 0.60) a				
Street food is tasty.	0.789 (fixed)	0.850	0.507	0.75
Street food is made fresh on the spot.	0.694 (9.955 ***)			
The temperature of street food is appropriate.	0.646 (9.443 ***)			
**Employee service** (3.15 ± 0.60)				
Vendor is friendly and always tries to help consumers.	0.807 (fixed)	0.900	0.594	0.81
Vendor is confident and knowledgeable about food.	0.718 (11.793 ***)			
Vendor reacts well to complaints or questions.	0.784 (12.782 ***)			
**Physical environment** (2.74 ± 0.76)				
Interior of street food stall is attractive.	0.775 (fixed)	0.867	0.629	0.84
Music of street food stall is enjoyable.	0.767 (12.524 ***)			
Lighting of street food stall is comfortable.	0.835 (13.341 ***)			
**Price** (3.21 ± 0.76)		0.896	0.751	0.89
The taste of the food is good compared to the price.	0.843 (fixed)			
The quantity of the food is good compared to the price.	0.896 (18.110 ***)			
The price of the food is reasonable.	0.843 (16.729 ***)			
**Rapidity** (3.77 ± 0.65)		0.933	0.725	0.84
The food is served quickly.	0.877 (fixed)			
The food can be easily purchased.	0.834 (11.356 ***)			
**Utilitarian Value** (3.96 ± 0.59)		0.914	0.626	0.83
I ate delicious food compared to the price.	0.791 (fixed)			
I achieved good value compared to the price.	0.801 (13.839 ***)			
Compared with what I expected to pay, the price was reasonable.	0.781 (13.472 ***)			
**Hedonic Value** (3.47 ± 0.62)		0.920	0.660	0.85
Street food consumption is enjoyable.	0.870 (fixed)			
Street food consumption makes me feel good.	0.859 (17.439 ***)			
Street food consumption is interesting.	0.697 (13.107 ***)			
**Repurchase Intention** (3.44 ± 0.71)		0.909	0.681	0.86
I will repurchase street food.	0.825 (fixed)			
I will revisit the street food stall soon.	0.790 (14.490 ***)			
I have the intention to eat street food often.	0.860 (15.806 ***)			

Note: (1) ^a^ SD = standard deviation; (2) *** *p* < 0.001; (3) CCR = composite construct reliability; AVE = average variance extracted. χ^2^(202) = 331.383 (*p* < 0.001); χ^2^/df = 1.641; goodness-of-fit Index (GFI) = 0.909; normed fit index (NFI) = 0.913; comparative fit index (CFI) = 0.963; root-mean-square-error of approximation (RMSEA) = 0.047.

**Table 3:** Correlation estimates.

	1	2	3	4	5	6	7
1. Food quality							
2. Employee Service	0.248						
3. Physical Environment	0.192	0.420					
4. Price	0.106	0.172	0.135				
5. Rapidity	0.155	0.086	0.314	0.228			
6. Utilitarian Value	0.227	0.314	0.240	0.219	0.227		
7. Hedonic Value	0.300	0.304	0.114	0.175	0.205	0.584	
8. Repurchase Intention	0.284	0.019	0.403	0.190	0.375	0.136	0.440

Note: matrix entries are the square correlations.

**Table 4:** Structural parameter estimates.

Hypotheses	Standardized Coefficients	t-Value	Results
H1 Food Quality → Utilitarian Value	0.207	2.419 *	Supported
H2 Employee Service → Utilitarian Value	0.158	1.981 *	Supported
H3 Physical Environment → Utilitarian Value	0.115	1.973 *	Supported
H4 Price → Utilitarian Value	0.279	5.531 ***	Supported
H5 Rapidity → Utilitarian Value	0.158	2.811 **	Supported
H6 Food Quality → Hedonic Value	0.333	3.723 ***	Supported
H7 Employee Service → Hedonic Value	0.161	2.031 *	Supported
H8 Physical Environment → Hedonic Value	0.121	2.091 *	Supported
H9 Price → Hedonic Value	0.117	2.423 *	Supported
H10 Rapidity → Hedonic Value	0.158	2.815 **	Supported
H11 Utilitarian Value → Repurchase Intention	0.359	4.113 ***	Supported
H12 Hedonic Value → Repurchase Intention	0.619	6.380 ***	Supported
Goodness-of-fit statistics	χ^2^(207) = 382.969 (*p* < 0.001) χ^2^/df = 1.850 GFI = 0.895 NFI = 0.899 CFI = 0.950 RMSEA = 0.055		

Note: GFI = goodness-of-fit index; NFI = normed fit index; CFI = comparative fit index; RMSEA = root-mean-square-error of approximation; * *p* < 0.05, ** *p* < 0.01, *** *p* < 0.001.

**Table 5:** Model fit indices of food safety risk groups.

		χ^2^	GFI	NFI	CFI	RMSEA	△χ^2^
Food safety risk groups	Configural invariance model	592.512	0.856	0.856	0.948	0.041	13.850
	Metric invariance model	606.362	0.853	0.852	0.948	0.040	

Note: △df = 15, △χ^2^ = 25.000 (*p* < 0.05).

**Table 6:** Moderating effects of food safety risk perception.

	Low-Risk Perception Group (N = 125)		High-Risk Perception Group (N = 165)		Unconstrained Model χ^2^ (df = 414)	Constrained Model χ^2^ (df = 415)	△χ^2^ (df = 1)
	S.E. ^(1)^	t-Value	S.E.	t-Value			
FQ → UV	0.328	3.034 **	0.020	0.196	653.633	658.683	5.050
FQ → HV	0.161	2.776 **	0.252	2.371 *	653.633	654.413	0.780

Note: ^(^^1)^ standard estimates; * *p* < 0.05, ** *p* < 0.01.; FQ = food quality, UV = utilitarian value, and HV = hedonic value.
